# Real-World Financial and Clinical Impact of Diagnostic-Driven and Empirical-Treatment Strategies in High-Risk Immunocompromised Patients with Suspected Aspergillus Infection in the United Kingdom

**DOI:** 10.1128/spectrum.00425-22

**Published:** 2022-05-09

**Authors:** Stephanie R. Earnshaw, Cheryl McDade, Andrew Bryan, Monica Ines, Christianne Micallef, Anita Sung, David A. Enoch

**Affiliations:** a RTI Health Solutions, Research Triangle Park, North Carolina, USA; b Pfizer Biopharmaceuticals Group, Pfizer Ltd., Surrey, United Kingdom; c Hospital & Vaccines Business Unit, Pfizer, Inc., Porto-Salvo, Portugal; d Cambridge University Hospitals NHS Foundation Trust, Cambridge, United Kingdom; e Pfizer, Inc., New York, New York, USA; University of Debrecen

**Keywords:** antifungal, aspergillosis, cost-effectiveness, economic evaluation, healthcare costs, invasive fungal infection

## Abstract

A diagnostic-driven (DD) treatment strategy has proven successful for treating invasive fungal infections (IFIs) caused by Aspergillus. However, uptake of this treatment strategy is not fully embraced. This study compares the economic and clinical impact of DD and empirical-treatment (ET) strategies used within hospitals. Methods: a decision-analytic model was developed to compare costs and clinical outcomes associated with ET or a DD strategy of identifying infections caused by Aspergillus via galactomannan-antigen testing or Aspergillus polymerase chain reaction (PCR) in neutropenic patients with unexplained fever. Patients were treated prophylactically with antifungal treatments as seen in United Kingdom (UK) hospitals. The IFI incidence, response, mortality, resource use, and adverse events were obtained from meta-analyses and other clinical studies. Analyses were performed from the U.K. hospital perspective, and costs were obtained from standard costing sources. Although diagnostic-testing costs increased, total cost and length of stay were reduced by £1,121 and 1.54 days when treating via a DD strategy. Intensive care and general ward days accounted for > 40% of total costs and > 58% of the cost reduction came from reduced antifungal costs. Treating with a DD strategy reduced the number of patients being treated with antifungal agents while survival was increased. Thus, a DD strategy was cost savings (-£136,787 cost per death avoided) compared with an ET strategy. Conclusion: this study suggests that incorporating a DD strategy as the preferred treatment protocol may be a cost-saving and clinically improved treatment strategy for managing neutropenic patients with unexplained fever.

**IMPORTANCE** Patients at risk of invasive fungal infections (IFIs), such as Aspergillus spp., tend to be immunocompromised and usually take several medications which may generate many side effects. Prescribing is further complicated by comorbidities, drug interactions and challenges accessing diagnostics. Therefore, adding another agent may be neither straightforward nor the best option for these types of patients. A diagnostic-driven (DD) treatment strategy has proven successful for treating IFIs. However, uptake of this treatment strategy is not fully embraced in clinical practice perhaps because this strategy is thought to be more costly and/or to result in higher mortality relative to treating empirically. We developed a decision-analytic model to examine the impact of these 2 strategies on costs and health outcomes. This study indicates that incorporating a DD strategy as the preferred treatment protocol may be a cost-saving and clinically improved treatment strategy for managing neutropenic patients with unexplained fever.

## INTRODUCTION

Aspergillosis, an invasive fungal infection (IFI) caused by the Aspergillus spp., is an infection with a high mortality in severely immunocompromised patients (1–3). The annual incidence has been found to range from 0.2 to 8.0% in various risk groups in the United Kingdom (UK) (4).

Patients are typically treated empirically with antifungal agents. In some cases, prophylaxis treatment is used. However, treatments are limited to those agents that are indicated for empirical use, meaning agents that are most effective in treating Aspergillus may not be used initially. In addition, patients at risk already tend to be on a large number of pharmacological agents where adding another agent may not be the best option for the patient.

A diagnostic-driven (DD) treatment strategy has proven successful for treating IFIs caused by Aspergillus in hematology patients (5–9). A DD strategy is one in which patients are tested and receive a positive test for infection with various fungal pathogens. Once the pathogen is identified, treatment can then be determined, and the best agent selected. However, treating via this approach is often thought of as being more costly because the use of expensive diagnostics is incurred and treating via empirical-treatment (ET) is thought to result in lower mortality because infections are less likely to be missed. Some hospitals which have adopted a DD strategy commence antifungals empirically and then review them once diagnostic tests are back.

Economic evaluations of the DD treatment strategy have been published that show that this treatment approach is both less costly and more effective (5–12). However, uptake of this treatment strategy is still not fully embraced despite growth in the population at risk, more appropriate treatment options becoming available, and real-world data to support a better understanding of the potential benefits from a reduced reliance on empirical treatment. This study compares the economic and clinical impact of DD and ET strategies used within a hospital while considering a more comprehensive treatment pathway, broader choice of antifungal treatments and their clinical risks and benefits, real-world resource use, and a broader set of outcomes.

## RESULTS

Out of 1,000 at-risk patients, 41 IFIs were estimated to occur; 109 IFIs would have occurred without prophylaxis. Use of a DD strategy was estimated to identify and treat 33 of those cases, whereas ET would have likely identified 10 cases. However, 57 patients would have been treated via ET.

Although increased costs occurred due to diagnostic testing for the DD strategy, per-patient costs related to antifungal agent use were higher in patients receiving ET and average patient total costs were reduced (£20,230 for DD versus £21,351 for ET). Days in the intensive care unit (ICU) and general ward accounted for > 40% of the total costs and > 58% of the cost reduction came from reduced antifungal costs. Given that survival among patients was similar (90.32% for DD and 89.50% for ET), a DD strategy was a cost-saving (less costly and more effective at -£136,787 per death avoided) strategy. Base-case results are presented in Table 1.

**TABLE 1:** Base-case results^*a*^

Model outcomes	Diagnostic-driven strategy	Empirical-treatment strategy	Incremental difference
Outcomes per 1,000 at-risk patients			
Total invasive fungal infections that exist	41	41	0.00
Total patients treated	33	57	−23.27
Total invasive fungal infections treated	33	33	0.00
Patients treated when invasive fungal infections exists and has been diagnosed	33	10	23.32
Total costs (per patient)	£20,230	£21,351	−£1,121
Antifungal and associated costs	£6,836	£7,517	−£441
ICU costs	£607	£662	−£55
General ward costs	£7,627	£8,239	−£612
Other medical costs	£5,160	£4,933	−£680
Total LOS (per patient)	19.13	20.68	−1.54
ICU days	0.40	0.44	−0.04
General ward days	18.73	20.24	−1.50
No. of patients dead (per 1,000 at-risk patients)	5.48	5.94	−0.46
Probability of survival	90.32%	89.50%	0.82%
No. needed to treat			122

a ICU = intensive care unit; LOS = length of stay.

### Sensitivity analysis.

**FIG 1:**
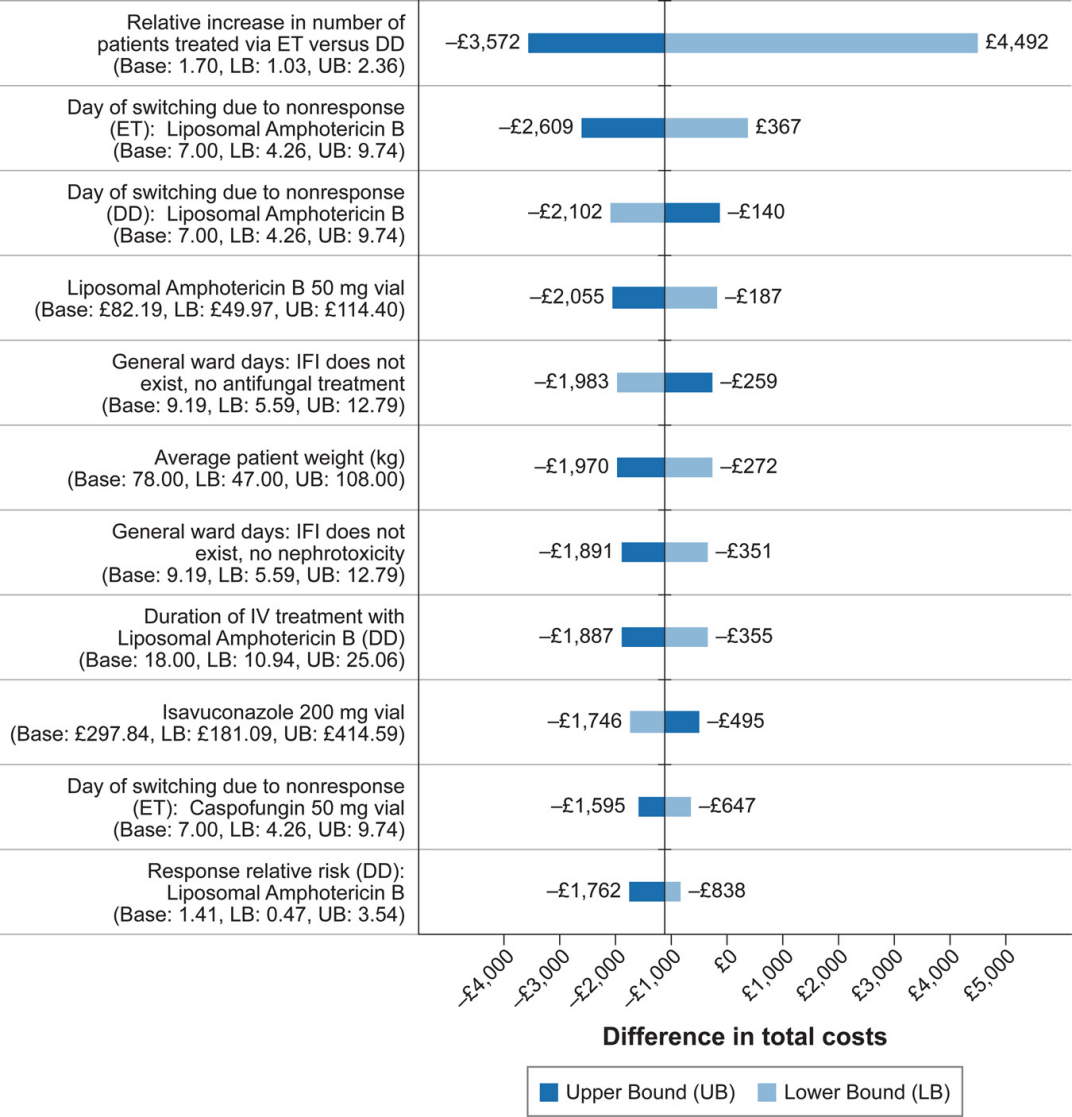
Impact on the difference in total cost when changing parameters one at a time.

One-way sensitivity analysis (Fig. 1) found the difference in total costs were most sensitive to changes in the relative increase in the number of patients treated empirically versus the DD strategy, such that ET became less costly when fewer patients were treated unnecessarily. The cost of treating via ET approached the cost of treating via a DD strategy when ET causes treatment of less than 1.5 times more patients.

Results were also sensitive to changes in the day at which switching occurs due to nonresponse to liposomal amphotericin B when treating empirically. In this situation, the sooner patients treated with ET could switch to caspofungin, the lower an ET patient’s costs became because patients were switched to a cheaper drug. This led us to perform a scenario analysis in which we compared the DD strategy with an ET strategy in which all patients were treated with caspofungin as a first-line antifungal agent. Although the cost of first-line caspofungin treatment was lower, ET with caspofungin was more costly (~£2,000) because caspofungin is not as efficacious. Changes in all other parameters within their plausible ranges (Fig. 1) did not affect the results in that the DD strategy remained less costly than the ET.

**FIG 2:**
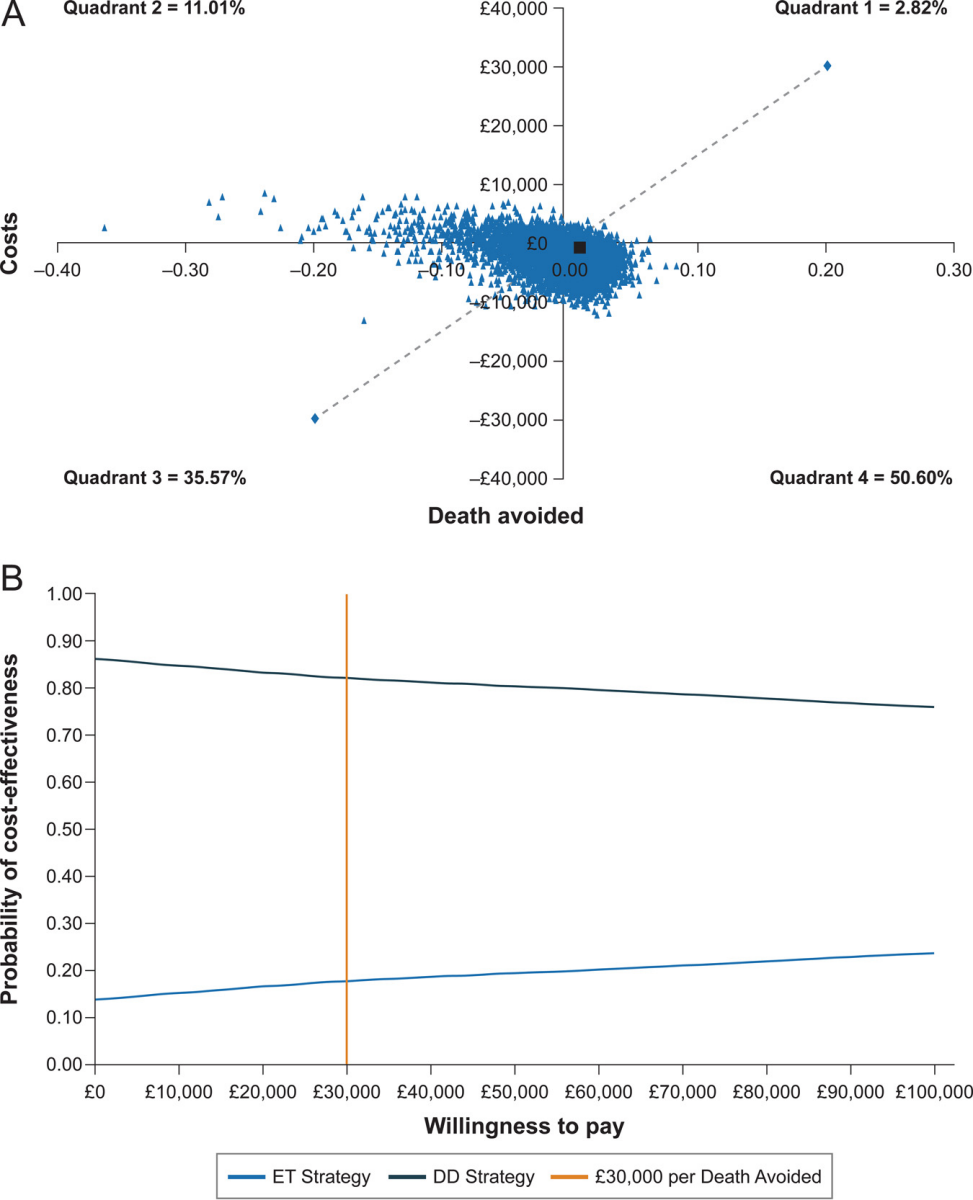
Probabilistic sensitivity analysis. A: Scatter plot. B: Cost-effectiveness acceptability curve. Dotted diagonal line represents an incremental cost per death avoided of £30,000. Black square represents the deterministic result.

Probabilistic sensitivity analysis (Fig. 2) showed that the DD strategy is less costly 86.17% of the time. The simulations support the conclusion that the DD strategy and the ET strategy have similar survival where 53.42% of the time survival is slightly better for patients treated via the DD strategy. The DD strategy was dominant 50.6% of the time, and the incremental cost per death avoided was cost-effective at £30,000 or less 82.22% of the time. However, these results should be interpreted with caution as the two approaches have similar survival. As a result, a good portion of the simulations fall within the third quadrant (35.57%) where the DD strategy is less costly and less effective.

## DISCUSSION

Although a DD strategy increases costs due to performing additional diagnostics upfront, a DD strategy reduces spending on antifungal agents. Further, overall costs are not increased and may even be slightly lower due to avoiding unnecessary treatment with costly antifungal agents. Given that this population of patients is already on a large number of pharmaceutical agents, a DD strategy could also reduce the pharmaceutical burden and possibly lead to fewer adverse events and drug-drug interactions that may not have been able to be fully accounted for within this analysis.

Along with this beneficial economic impact, patient survival seems to be similar among patients treated with DD and ET because patients are better selected for treatment (i.e., patients with infection receive more efficacious agents while avoiding unnecessary treatment), which is consistent with the clinical trials that examine the efficacy of a DD strategy. In addition, the DD strategy has the potential to free up beds in both the ICU and general ward for other patients, reducing burden to the overall health care system.

Several economic evaluations of treatments for IFIs have been published. Most focus on comparing treatment of IFIs with one antifungal agent versus another antifungal agent within their respective indications (i.e., empirical therapy or known infection) (12–16). Few have focused on the comparison of treatment using a DD versus an ET strategy (10, 11, 17, 18). This analysis differs from Macesic et al. (18) in that it attempts to mimic real-world practice in the UK whereas Macesic et al. used trial-based costing with an Australian perspective and United States-based costs (18). Although this analysis is similar to the Barnes et al. (10) and Mao et al. (11) analyses, there are important differences that make this analysis valuable. Among a few of the importance differences are the incorporation of additional antifungal agents. Over the past several years, new antifungal agents (i.e., posaconazole and isavuconazole) have been approved for use in this population of patients. This analysis includes the real-world use of those antifungal agents and a revised treatment pathway by including second-line treatment and a more comprehensive impact of prophylaxis. With this, we have incorporated the clinical impact from various comparative effectiveness studies that have since been published (19–21). Furthermore, we have been able to capture more of a real-world impact by incorporating resource use seen in actual U.K. clinical practice (22) and LOS in the ICU and general ward.

Although this analysis considers more comprehensive treatment pathways and is more representative of actual clinical practice, it is not without limitations. Specifically, ICU and general ward days given different clinical outcomes (i.e., IFI exists/does not exist, and nephrotoxicity occurs/does occur) were estimated from the SECURE clinical trial and enhanced with information from various published studies rather than obtained for each patient type. It would be preferable to be able to include estimates of LOS that may have been seen in actual clinical practice; however, LOS was found to not be a key driver of cost differences between the different treatment strategies. As a result, we believe that results would not be impacted much with more refined estimates.

The model assumptions for general IFI incidence, overall mortality and IFI mortality were taken from the experience of a single tertiary care center in Germany over 1995–2006 (23), and therefore might not reflect the experience of other centers, nor reflect the benefits accrued from subsequent improvements in wider patient management. However, it was felt this was balanced by the need to use data from a period before the widespread use of triazole agents for prophylaxis to avoid double counting the benefits of prophylaxis in this analysis. Furthermore, the incidence of IFI in the earlier study (10.9%) is consistent with that seen in a meta-analysis of prophylaxis clinical studies published in 2016 in which the reported average incidence was 12.5% (19).

Although resource use within the model was obtained from actual clinical practice, they were obtained from a single hospital site in the UK, which might not represent resource use seen in other hospitals. Further, these data were based on treatment as seen in 2008 to 2010. A number of new antifungal treatments have been approved over the past several years, which could impact actual resource use. However, the data from Ceesay et al. (22) were comprehensive and did demonstrate differences when patients had proven/probable versus no evidence of IFI. This evidence is more than what has been seen in previous publications.

The model allows for multiple tests (galactomannan [GM], polymerase chain reaction [PCR], and β-D glucan) to be given. However, the sensitivity/specificity of tests given in combinations and/or in a series are not well published. As a result, it may be questionable whether the accuracy of the diagnostics is accounted for appropriately. The sensitivity of diagnostic tests affects the number of patients who are ultimately deemed to have an IFI and to be treated. The sensitivity of tests is also affected by the use of prophylactic, mold-active azoles (i.e., posaconazole). We believe that when treating patients, physicians will err on the conservative side of treatment, such that if any of the tests resulted in a positive, patients would be treated with antifungal agents indicative of an IFI existing. Thus, the results of this analysis would not be that far from what may be seen in actual clinical practice. We assumed that the DD strategy and ET were initiated at the same time in our model. We did this as we noted that just because a DD strategy is incurred, it does not mean that patients have to wait to be treated. As such, we incorporated this clinical recommendation into the analysis.

In conclusion, a DD strategy may be able to more efficiently identify patients who are likely to benefit from IFI treatment in a population already exposed to multiple drug therapies (i.e., reduce the pharmaceutical burden and possibly lead to fewer adverse events) and reduce bed days. Although there were increased costs due to the use of diagnostic assays, incorporating a DD strategy as the preferred treatment protocol may be cost-saving and may improve survival for managing patients with neutropenia with unexplained fever.

## MATERIALS AND METHODS

We adapted a previously developed decision-analytic model to compare the cost and outcomes that may be experienced by patients treated via ET and a DD strategy based on typical antifungal use (10, 11).

The model was developed from a U.K. perspective with a time horizon of 6 months. Details are available in the Supplementary Material. Good modeling practices guidelines and the Consolidated Health Economic Evaluation Reporting Standards (CHEERS) were followed (24–26).

### Patient population.

Patients were severely immunocompromised adults (18 years of age or older), such as those with hematological malignancies scheduled for chemotherapy or autologous/allogeneic stem-cell transplantation. Patients were expected to become neutropenic (neutrophil count < 500 cells/mm^3^) for at least 10 days (5–9).

### Comparators.

We compared the impact of treating patients using 2 treatment strategies as seen in actual clinical practice in the UK.

**DD treatment.** Patients initiate antifungal treatment with confirmation of IFI through clinical assessment, Aspergillus colonization, and/or enzyme-linked immunosorbent assay (ELISA) positive results identified through bronchoalveolar galactomannan (BAL-GM), serum GM, PCR, or β-d-glucan test and positive or abnormal computed tomography (CT) scan and fever. DD treatment is started just as early as ET. Patients treated via a DD strategy are treated with caspofungin (15.8%), isavuconazole (12.2%), voriconazole (12.7%), or liposomal amphotericin B (59.3%) (27–32).

**ET.** Patients initiate antifungal treatment when they are suspected of having an IFI (i.e., when they had persistent and/or recurrent fever and were unresponsive to antibacterial therapies) but do not have further confirmation of the existence of an IFI. Patients treated via ET are treated with liposomal amphotericin B (65.8%) or caspofungin (34.2%) (27, 28, 31).

### Model structure.

**FIG 3:**
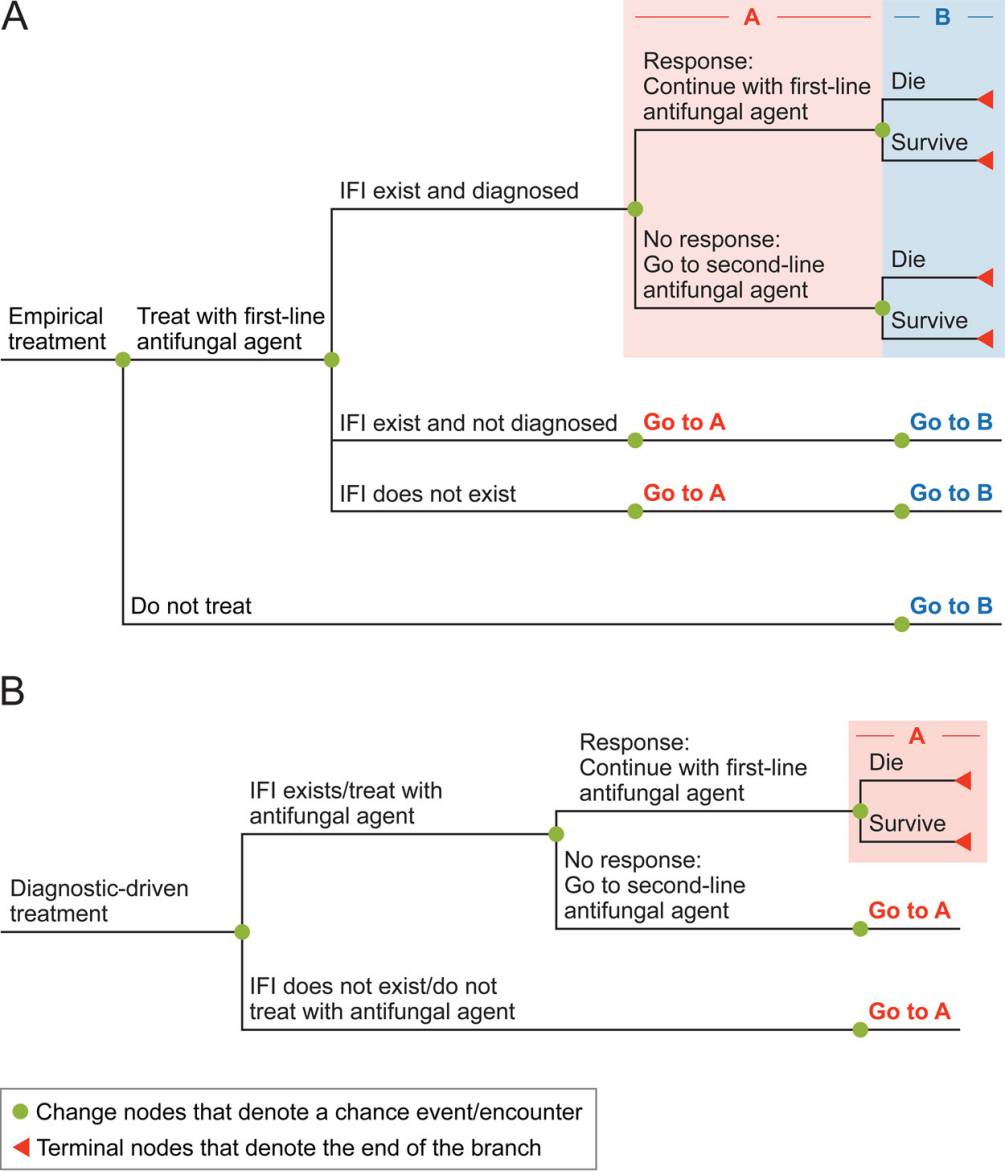
Model structure. A: Empirical treatment. B: Diagnostic-driven treatment.

Patients with neutropenia who are at risk for IFI enter the decision tree model (Fig. 3). Patients receive standard clinical management of blood cultures, imaging, and other monitoring and microbiological tests, as indicated. These patients also receive broad-spectrum antibiotics and are treated prophylactically with posaconazole.

At a clinically appropriate time, a decision is made to treat this cohort via a DD strategy or an ET strategy. Patients receiving ET (Fig. 3A) are treated with a first-line antifungal agent on the basis of clinical suspicion of aspergillosis (i.e., patients have/don’t have the existence of IFI confirmed). Responding patients continue treatment with this antifungal agent until the IFI resolves or death. Nonresponding patients switch to a second-line antifungal agent. Patients continue this treatment until the IFI resolves or death. In this arm of the decision tree, patients may have an IFI and it is diagnosed, have an IFI and it never be diagnosed, or may not have an IFI at all.

In the DD strategy (Fig. 3B), the decision to treat with an antifungal agent is based on a confirmed diagnosis of an IFI from clinical and biomarker findings. Patients without a confirmed diagnosis receive optimal care without antifungal agents. Patients with a confirmed diagnosis either respond or do not respond to a first-line antifungal agent. Based on response, patients receive a second-line antifungal agent or not. The IFI either resolves or the patient will die as a result.

Patients in the DD strategy are treated similarly to patients in ET, except that patients treated with a DD strategy receive additional health care resources in order to diagnose the IFI. It is assumed that the DD strategy and ET are initiated at the same time.

### Model inputs.

**(i) Incidence of IFIs.** Incidence of IFIs of 10.9% (95% confidence interval [CI], 9–13%) was obtained from a retrospective cohort study designed to evaluate the IFI incidence and mortality in patients at risk for IFIs (23). As seen in the DD and empirical comparative clinical studies, ET identified 29% to 31% of the IFIs that the DD strategy confirmed (5, 9). We assumed 1.6987 times more patients are treated when treating via ET (5, 33, 34).

**(ii) Sensitivity of DD tests.** The sensitivity of the diagnostic tests, such as PCR, BAL-GM, serum GM, and β-d-glucan (i.e., proportion of positives that are correctly identified), was considered in the model. Patients in the DD strategy received multiple tests to identify IFI. These tests may occur in combination or in sequence with each other. Because the sensitivity of combination and sequentially given tests for identifying IFI are limited in the literature, we assume sensitivity based on a single test. Sensitivity in this analysis was assumed to be 0.82 using PCR in the base case (35). We examine the impact of changes in sensitivity in sensitivity analysis.

**(iii) Prophylaxis.** Patients were treated prophylactically with posaconazole, which impacts the incidence of IFI. The odds of an IFI relative to no treatment was 0.13 (95% CI, 0.05–0.30) (19). The odds ratio was converted to relative risk. Prophylaxis treatment was given for 8.0 (4.86–11.14) days (5).

**(iv) Response.** Odds of response for patients on antifungal agents when patients have an IFI were obtained from a meta-analysis (20). From these data, the relative risk of response and the percentage of patients responding to treatment was calculated (Table 2).

**TABLE 2:** Clinical inputs: base case (range)^*a*^

Clinical effect	Odds of response: IFI exists^*b*^ (relative to isavuconazole)	Odds of response: ET and given no IFI strategy^*c*^ (relative to amphotericin B)	Mortality for patients with IFI when treated with antifungal agents^*d*^ (relative to isavuconazole)
Percentage responding	0.35	0.39	0.30
Caspofungin	−0.99 (95% CI, −2.21 to 0.29)	0.72 (95% CI, 0.38 to 1.29)	0.32 (95% CI, −0.19 to 0.84)
Isavuconazole	—^*g*^	0.92 (95% CI, 0.43 to 1.76)^*h*^	—^*g*^
Liposomal amphotericin B	−0.99 (95% CI, −2.21 to 0.29)	0.80 (95% CI, 0.52 to 1.24)	0.18 (95% CI, −1.17 to 1.52)
Voriconazole	0.06 (95% CI, −0.43 to 0.57)	0.92 (95% CI, 0.43 to 1.76)	0.32 (95% CI, −0.19 to 0.84)

Adverse events^*e*^	Hepatotoxicity	Hypertension	Nephrotoxicity^*f*^	Tachycardia
Liposomal amphotericin B	14.21%	0.60%	12.13%	0.60%
Caspofungin	12.01%	0.60%	3.23%	0.60%
Isavuconazole	6.94%	0.60%	11.53%	0.60%
Voriconazole	8.82%	0.60%	11.53%	0.60%

a CI = confidence interval; ET = empirical treatment; IFI = invasive fungal infection.

b Herbrecht et al. (20) and Maertens et al. (39) caspofungin response was assumed to be similar to liposomal amphotericin B (21).

c Chen et al. (21) estimated as weighted average of Boogaerts et al. (47), Schuler et al. (48), and Herbrecht et al. (20).

d Herbrecht et al. (20) and Maertens et al. (39) overall mortality for caspofungin was assumed to be similar to overall mortality experienced by patients on voriconazole as it was considered a novel antifungal agent in Hahn-Ast et al. (23).

e Walsh et al. (36), Walsh et al. (37), Walsh et al. (38), and Maertens et al. (39).

f Amphotericin B trials defined as a doubling of the serum creatinine level or an increase of at least 1 mg/dL (88 μmol/L) if elevated at baseline. (36) Caspofungin defined as increase in total bilirubin for caspofungin. (38) Isavuconazole was taken from Maertens et al. (39). Voriconazole defined as > 1.5 times x baseline of serum bilirubin during therapy for voriconazole. (37).

g —, this is the reference drug. As a result, there is no odds relative to itself.

h Isavuconazole was found to be similar in efficacy to voriconazole in the SECURE trial. Response given empirical treatment assumed similar to voriconazole (39).

Response for patients with no proven IFI was calculated using data from the ET trials. Specifically, odds of response for patients on ET was obtained from a network meta-analysis of ET trials (21). These data were then used to calculate response given no IFI (Table 2), which differs depending upon which antifungal agent the patient received.

**(v) Second-line treatment.** Patients who do not respond to first-line treatment switch to second-line treatment after 7 days of first-line treatment, consistent with clinical opinion (C. Micallef and D. Enoch, Cambridge University Hospitals NHS Foundation Trust, personal communication). In the DD strategy, patients treated with liposomal amphotericin B switch to isavuconazole. Patients treated with voriconazole, isavuconazole, or caspofungin switch to liposomal amphotericin B. This is consistent with recent cost-effectiveness analysis in patients with invasive aspergillosis (13–15).

In ET, patients treated with liposomal amphotericin B switch to caspofungin, while patients treated with caspofungin switch to liposomal amphotericin B.

**(vi) Mortality.** For patients at risk for IFIs, clinical success is typically defined as surviving treatment. As such, base mortality or mortality for patients with an IFI/on amphotericin B treatment and for patients without an IFI are calculated based on a retrospective, real-world cohort of high-risk patients (23). Details are presented in the Supplementary Materials. Relative risk adjustments to mortality for patients in whom IFI exists while being treated with other antifungal agents were obtained from a meta-analysis (Table 2).

**(vii) Adverse events.** Adverse events were limited to hypertension, hepatotoxicity, nephrotoxicity, and tachycardia (i.e., those that tended to occur in 10% or more of patients and/or were deemed to be clinically significant or resource intensive) (C. Micallef and D. Enoch, Cambridge University Hospitals NHS Foundation Trust, personal communication). The percentage of patients experiencing adverse events were obtained from the clinical trials (Table 2) (36–39).

**(viii) Resource use and costs.** The dosing for each antifungal agent was obtained from the U.K. summary of product characteristics (SmPC) for each medicine and confirmed by the key opinion leaders (C. Micallef and D. Enoch, Cambridge University Hospitals NHS Foundation Trust, personal communication) (28–31, 40–42). Patients are first treated with intravenous (IV) therapy and then stepped down to oral therapy with the same treatment or with posaconazole if the IV treatment does not have an oral formulation. An average patient weight of 77.91 kg is assumed for costing (43). Dosing and administration and acquisition costs are presented in Table 3.

**TABLE 3:** Antifungal agent resource use and costs: base case (range^*a*^)

Antifungal	Antifungal costs^*b*^	Administration time^*c*^	Duration of treatment^*d*^	Therapeutic drug monitoring^*e*^
Caspofungin 70 mg vial	£52.91	1.00	18 (Interquartile, 13, 34)	NA
Caspofungin 50 mg vial	£42.90	1.00	18 (Interquartile, 13, 34)	NA
Isavuconazole 200 mg vial	£297.84	1.00	8.50 (SD, 9.05)	1.00
Isavuconazole 100 mg tablet	£42.81	NA^*f*^	38.25 (SD, 23.20)	1.00
Liposomal amphotericin B 50 mg vial	£82.19	1.00	18 (Interquartile, 13, 34)	NA
Voriconazole 200 mg vial	£77.14	2.0 (day 1), 1.5 (day 2+)	8.50 (SD, 9.05)	1.00
Voriconazole 200 mg tablet	£22.64	NA	38.25 (SD, 23.20)	1.00
Posaconazole 100 mg tablet	£2.39	NA	Prophylaxis: 8 step down treatment: 44.44	1.00

a Estimates with no identified range used ± 20%. Dashes indicate not applicable. SD = standard deviation.

b International Institute for Health Care and Excellence (2020) (49).

c Fungizone SmPC (2019) (50), CANCIDAS SmPC (2020) (28), CRESEMBA SmPC (2020) (29), AmBisome Liposomal SmPC (2018) (31), and VFEND SmPC (2020) (30).

d Marty et al. (51) and Horn et al. (52).

e Ashbee et al. (44).

f NA, not applicable.

Durations of IV and oral antifungal treatment were estimated from a variety of clinical studies (Table 3). Because of the limited availability of data, the duration of treatment for patients treated via DD strategy or ET was assumed to be the same. Patients switching to second line are assumed to have 7 days of IV treatment of their first-line antifungal treatment. Once they switch, they incur the full duration of treatment as if they were successful on first-line treatment.

Patients on azoles are subjected to therapeutic drug monitoring. The frequency of monitoring was obtained from Ashbee et al. (44) and is reported in Table 3.

The model considers length of stay (LOS) in terms of general ward and intensive care unit (ICU) days. It further distinguishes LOS by whether patients have an IFI with nephrotoxicity, an IFI without nephrotoxicity, or no IFI as LOS is likely to differ by these outcomes. The LOS by patient type is presented in Table 4 and details are presented in the Supplementary Materials.

**TABLE 4:** Other medical resource use and costs^*a*^

Diagnostic tests^*b*^	Percentage receiving^*c*^	No. resources no evidence of IFI^*c*^	No. resources no evidence of IFI^*c*^	Cost per unit^*d*^
Aspergillus galactomannan antigenemia test (serum or BAL)	100.00%	27.00	9.00	£117.30
Aspergillus PCR test	100.00%	2.00	0.00	£231.34
β-D-glucan test	100.00%	3.00	1.00	£77.85

Resources	Resource use when IFI exists		Resource use when no evidence of IFI		Cost per unit^*d*^
	Percentage receiving^*c*^	No. of resources^*c*^	Percentage receiving^*c*^	No. of resources^*c*^	
Chest X-ray	83.33%	4.00	83.33%	1.00	£120.83
Blood culture	100.00%	17.00	100.00%	4.00	£3.71
Testing for hypomagnesaemia	87.78%	30.00	100.00%	12.00	£1.10
Nasal, pharyngeal, and rectal swab	100.00%	30.00	100.00%	12.00	£1.10
CT scan (one area, no contrast)	100.00%	4.00	100.00%	1.00	£77.95
Full blood count	100.00%	28.00	100.00%	16.00	£6.28
Liver function test	100.00%	28.00	100.00%	16.00	£1.76
C-reactive protein test	100.00%	25.00	100.00%	1.00	£1.76
Renal function test	100.00%	30.00	100.00%	12.00	£6.28
Bronchoscopy	25.00%	1.00	18.00%	1.00	£787.14
Bronchoalveolar lavage	25.00%	1.00	18.00%	1.00	£787.14
Lung biopsy	2.00%	1.00	2.00%	1.00	£967.40
Outpatient visits	100.00%	5.00	100.00%	4.00	£166.51
Antibiotics	100.00%	120.00	100.00%	23.00	£116.63
avg LOS in hospital for resources above (days)		120.00		57.00	

LOS^*e*^	ICU days	General ward days	Total LOS
IFI exists, nephrotoxicity	0.70	28.39	29.09
IFI exists, no nephrotoxicity	0.30	19.09	19.39
IFI does not exist, antifungal treatment and nephrotoxicity	0.50	13.43	13.93
IFI does not exist, antifungal treatment with no nephrotoxicity	0.10	9.19	9.29
IFI does not exist, no antifungal treatment	0.10	9.19	9.29

Adverse events^*f*^	Cost (£)
Hypertension	£1,054.82
Tachycardia	£1,135.88
Nephrotoxicity	£58.20
Hepatotoxicity	£73.23

a BAL = bronchoalveolar lavage; CT = computed tomography; ICU = intensive care unit; IFI = invasive fungal infection; LOS = length of stay; PCR = polymerase chain reaction. Range of ± 20% used in sensitivity analyses for all parameters.

b Resource use was normalized to the LOS used our analysis.

c Ceesay et al. (22) and personal communications with C. Micallef and D. Enoch (2021).

d NHS (2020) (53), NICE (2017) (54), NICE (2020) (55), Curtis and Burns (2019) (56), and Talent.com (2021) (57).

e Horn et al. (52), Ceesay et al. (22), and Bruynesteyn. (13).

f EASL (2019) (58), Curtis and Burns (2019) (56), NHS (2020) (53), Resuscitation Council (UK) (2015) (59), International Institute for Health Care and Excellence (2020) (49), NICE (2019) (60), and personal communications with C. Micallef and D. Enoch (2021).

The number, frequency, and types of diagnostic tests administered for the DD strategy and the number, frequency, and types of other health care resources administered for patients in whom IFIs exist/do not exist were obtained from a U.K. study in which the resource use of patients with suspected IFI was obtained (22). These data were adjusted for patients’ respective LOS and are presented in Table 4.

Costs for treating adverse events were based on resource use as recommended by published treatment guidelines, where available, and are supplemented by key opinion leaders’ input (Table 4) (C. Micallef and D. Enoch, Cambridge University Hospitals NHS Foundation Trust, personal communication).

All costs were presented in 2020 British pounds sterling. Value added tax (VAT) of 20% was included for administration of all inpatient drugs (45). Additional details on resource use and costs used within the model is presented in the Supplementary Materials.

### Model analysis.

Outcomes included the number of IFIs that exist in addition to the number of patients treated, IFIs treated, and IFIs identified and treated. The LOS in the ICU, LOS in the general ward, and patient survival were also estimated. Costs in terms of administration, acquisition for antifungal prophylaxis and treatment, and other health care resources were calculated.

The incremental cost per death avoided was calculated as follows:

= (C1−C2)÷(Q1−Q2)

where C1 is total cost incurred when treated via the DD strategy, C2 is total cost incurred when treated via ET, Q1 is total survival when treated via the DD strategy, and Q2 is total survival when treated via ET.

### Sensitivity analyses.

To test the robustness of the model assumptions and specific parameters used in the analysis, we examined the effect of changing parameters and assumptions in one-way sensitivity analyses.

Probabilistic sensitivity analyses (second-order Monte Carlo simulation) were also performed. Analyses were run 10,000 times to ensure stability in the results.

### Data availability.

A decision model was developed which compiled data from published sources to generate results.
