# Supplementary material for: Real-World Financial and Clinical Impact of Diagnostic-Driven and Empirical-Treatment Strategies in High-Risk Immunocompromised Patients with Suspected Aspergillus Infection in the United Kingdom
Source: Microbiol Spectr. 2022 May 9;10(3):e00425-22. doi: 10.1128/spectrum.00425-22 (PMC9241825; doi:10.1128/spectrum.00425-22)
Supplement: SUPPLEMENTAL FILE 1 — Supplemental material. Download spectrum.00425-22-s001.pdf, PDF file, 0.2 MB [file spectrum.00425-22-s001.pdf]

Real-world financial and clinical impact of diagnostic-driven and empirical-treatment strategies in high-risk immunocompromised patients with suspected *Aspergillus* infection in the United Kingdom

Supplementary Material

MATERIALS AND METHODS

Model Inputs

Prophylaxis

We used the impact on incidence of invasive *Aspergillus* and invasive *Candida* infections because the data are more robust, and the authors noted that treatment effects of posaconazole versus voriconazole for invasive aspergillosis should be interpreted with caution.[1]

The odds ratio of invasive fungal infection (IFI) relative to no treatment was converted to a relative risk using the formula

$$RR = OR / (1 - OR) + (OR * \text{incidence of IFI})$$

where RR is the relative risk of posaconazole relative to no treatment and OR is the odds ratio of posaconazole relative to no treatment. Relative risks are versus no treatment. We assumed the base incidence in the model includes non–mould-active prophylaxis of fluconazole. As a result, the impact to incidence of IFI was calculated relative to fluconazole.

Incidence of IFI is consistent with the incidence reported by Cornely et al. (2007), which reported on a randomised, controlled trial of the use of azole (fluconazole, itraconazole, and posaconazole) prophylaxis in which the incidence of IFI was estimated at any time during the 12-week treatment period.[2]

Response

The odds ratios for patients on antifungal agents when patients have IFI were versus isavuconazole. The odds ratios of response versus isavuconazole were converted to a relative risk using the formula

$$RR = OR / (1 - OR) + (OR * \text{isavuconazole response}),$$

where RR is the relative risk of treatment A relative to isavuconazole response of 0.35 and OR is the odds ratio of treatment A relative to isavuconazole response. Relative risks were then multiplied by isavuconazole response in order to derive response for the respective treatments.

Response for patients with no proven IFI was calculated from the response odds ratios for patients receiving empirical treatment (ET). Relative risks were calculated using the formula above where the odds ratios were versus use of amphotericin B. Response for patients with no proven IFI was then calculated by solving this formula:

$$R_{ET} = \text{incidence of IFI} * R_{IFI,A} + (1 - \text{incidence of IFI}) * R_{N,A},$$

where $R_{ET,A}$ = response to treatment A given ET, $R_{IFI,A}$ = response to treatment A given proven IFI, and $R_{N,A}$ = response to treatment A given no proven IFI.

Mortality

A base mortality (overall mortality in patients treated with amphotericin B) was estimated from a retrospective cohort study designed to evaluate IFI incidence and overall and IFI mortality over time in patients at risk for IFI.[3] Ideally, we would extract mortality from head-to-head clinical studies. However, the clinical studies had design issues that prevented us from being able to extract data from these studies. These included patients were treated with a mix of antifungal agents, rather than with select antifungal treatment, and overall survival was very high, which may have been influenced by study eligibility criteria.[4-6] As a result, the impact on survival in actual clinical practice was difficult to ascertain. Calculation of mortality seen in patients treated with amphotericin B in a retrospective, real-world study is presented in Table A-1. This

population was made up of neutropenic patients treated with myelosuppressive chemotherapy for haematological malignancies or autologous stem-cell transplantation.

Resource Use and Costs

Length of stay (LOS) was derived from a number of studies. A baseline total LOS of 19.90 days (standard deviation, 23.80) (average of 19.7 days for isavuconazole and 20.10 days for voriconazole) for patients with IFI was obtained from a within-trial economic analysis of SECURE.[7] These data are for patients with and without nephrotoxicity. The total LOS for patients without IFI was estimated assuming the ratio of total LOS for patients with/without IFI in a retrospective, medical chart abstraction study performed in patients in the United Kingdom (UK) with possible IFI.[8]

Patients who had nephrotoxicity had a total LOS 1.5 times the total LOS experienced by patients without nephrotoxicity.[9] Intensive care unit (ICU) days were obtained from this same economic evaluation, which align with actual clinical practice.[8,9] ICU days were assumed to be a portion of the overall LOS.

Derivation of unit costs are presented in Table A-2. Treatments for adverse events were as noted in the published treatment guidelines, where available, supplemented by key opinion leaders' input (Table A-3) (C Micallef and D Enoch, Cambridge University Hospitals NHS Foundation Trust, personal communication). Adverse events were assumed to be treated up to the time of the switch to a different antifungal agent.

Model Analysis

For one-way sensitivity analysis, individual parameters and assumptions varied based on 95% confidence intervals (CIs), plausible ranges from the literature, or ± 20% when neither CIs nor plausible ranges were available. Results were plotted on tornado diagrams in which inputs were presented from most sensitive to least sensitive.

In probabilistic sensitivity analyses, the percentage of patients using various healthcare resources, baseline response, IFI-related mortality, overall mortality, percentage of IFIs identified, diagnostic sensitivity, and percentage of patients experiencing adverse events were varied according to a beta distribution. The percentage of patients receiving the various antifungal treatments in each approach was drawn from a Dirichlet distribution. All other parameters were drawn from a gamma distribution.

Table A-1. Mortality for Patients With IFI on Amphotericin B or Without IFI

Parameter	Model Estimate
Overall mortality	10.7% (95% CI, 9%-13%)
IFI incidence	10.9%
IFI-related mortality	28.6% (95% CI, 19%-39%)
Calculated mortality of patients without IFI (may be on an amphotericin-based treatment, novel treatment, or no treatment)[a]	7.6%
Percentage of patients treated with novel antifungal agents[b]	17.86%
Hazard ratio of improvement in overall survival[c]	0.589 (95% CI, 0.362-0.959)
Calculated mortality of patients with IFI when treated with amphotericin B[d]	40.4%

CI = confidence interval; IFI = invasive fungal infection.

Source: Hahn-Ast et al.[3]

[a] Calculated as overall mortality = incidence of IFI × (X + IFI-related mortality) + percentage of patients without IFI × X, where X is the mortality of patients without IFI. Mortality of patients without IFI is assumed to be the same regardless of what IFI treatment patients receive because antifungal agents are assumed to affect only IFI mortality.

[b] Within Hahn-Ast et al., 15 of 84 patients were noted as being on novel antifungal agents.[3]

[c] Multivariate analysis of overall survival in patients with IFI who reported the use of novel antifungal agents was associated with a significant improvement in overall survival.

[d] Calculated as mortality of patients with IFI on a mix of antifungal agents (amphotericin-based and novel treatment) = percentage of patients on novel antifungals * (1-0.589) * X + (100% − percentage of patients on novel antifungals) * X where X = mortality of patients with IFI when treated with amphotericin B.

Table A-2. Unit Cost Details

Resource Use	Unit Cost	Source/Assumption
Aspergillus galactomannan antigenemia test (serum or BAL)	£117.30	NICE (2017)[10] reports "Unit costs of microbiological tests at a reference laboratory range from £55 to £59 per patient sample.[10] The cost per hour of a band 5 healthcare or biomedical scientist, who would carry out the text, is £33 to £35".[10] Cost of a scientist to perform the test was from the latest PSSRU.[11] Medical courier fees were included using the hourly wage for a medical delivery driver.[12] We assumed 2 hours per test.
Aspergillus PCR test	£231.34	Average of PCR costs from NICE guidance.[13] The cost of scientist to perform the test was from PSSRU.[11] Medical courier fees were included using the hourly wage for a medical delivery driver.[12] We assumed 2 hours per test.
β-D-glucan test	£77.85	Test cost obtained from NICE.[10] Cost of a scientist to perform the test was from PSSRU.[11] Medical courier fees were included using the hourly wage for a medical delivery driver.[12] We assumed 2 hours per test.
Chest x-ray	£120.83	IMAG - RD01A - direct access - Scan of One Area, without Contrast, 19 years and over[14]
Blood culture	£3.71	DAPS - DAPS08 - Phlebotomy[14]
Testing for hypomagnesaemia	£1.10	DAPS - DAPS04 - Clinical Biochemistry[14]
Nasal, pharyngeal, and rectal swab	£1.10	DAPS - DAPS04 - Clinical Biochemistry[14]

Resource Use	Unit Cost	Source/Assumption
CT scan (one area, no contrast)	£77.95	IMAG - RD20A - direct access - Computerised Tomography Scan of One Area, without Contrast, 19 years and over[14]
Full blood count	£6.28	DAPS - DAPS09 - Other Pathology Services[14]
Liver function test	£1.76	DAPS03 - direct access - Integrated Blood Services[14]
C-reactive protein test	£1.76	DAPS03 - direct access - Integrated Blood Services[14]
Renal function test	£6.28	DAPS - DAPS09 - Other Pathology Services[14]
Bronchoscopy	£787.14	DZ69A Diagnostic Bronchoscopy, 19 years and over. This is the average cost of elective inpatient, elective inpatient excess bed day, non-elective inpatient long stay, non-elective inpatient long stay excess bed day, non-elective inpatient short stay, day cases, outpatient procedures, regular day/night admissions[14]
Bronchoalveolar lavage	£787.14	Assumed to be the same as bronchoscopy.
Lung biopsy	£967.40	Total HRG's - DZ71Z - Minor Thoracic Procedures[14]
Outpatient visit	£166.51	NHS Ref cost 2018/19 - Service code 303 - Clinical Haematology[14]
Antibiotics	£116.63	Antibiotics are gram-negative such as co-trimoxazole (C Micallef and D Enoch, Cambridge University Hospitals NHS Foundation Trust, personal communication). Co-trimoxazole: £47.15 for 10 ampoules.[15] Administration Co-trimoxazole SmPC (2020)[16]: VAT included for IV administrations in hospital.[17]

Resource Use	Unit Cost	Source/Assumption
General ward (cost per day)	£407.17	SA17H - Total HRGs: Non-elective inpatients, long stay, unspecified acute lower respiratory infection (weighted average of DZ22M, DZ22N, DZ22P)[14]
ICU (cost per day)	£1,504.47	Non-specific, general adult critical care patients predominate (Total costs)[14]
Day ward nurse hourly wage	£47.00	PSSRU - Hospital-based nurses - Band 6 - cost per working hour £47, working time = 42 weeks (1,573 hours) per year, 37.5 hours per week[11]
Biomedical scientist hourly wage to perform diagnostic tests	£38.00	PSSRU - Hospital-based nurses - Band 5 healthcare or biomedical scientist - cost per working hour £38, working time = 42 weeks (1,573 hours) per year, 37.5 hours per week[11]
Courier hourly wage	£11.15	Talent.com (2021)[12]
Therapeutic drug monitoring cost (per test)	£65.49	NHS (2020)[18]

BAL = bronchoalveolar lavage; BNF = British National Formulary; CT = computed tomography; HRG = healthcare resource group; ICU = intensive care unit; IV = intravenous; NHS = National Health Service; NICE = National Institute for Health and Care Excellence; PCR = polymerase chain reaction; PSSRU = Personal Social Services Research Unit.; SmPC = summary of product characteristics; UK = United Kingdom; VAT = value-added tax.

Table A-3. Costs of Treatment for Adverse Events

Adverse Event	Adverse Event Cost		Source/Assumptions
Hypertension	£1,054.82		NICE (2019)[19]; C Micallef and D Enoch, Cambridge University Hospitals NHS Foundation Trust, personal communication
**Components**	**Unit Cost**	**Number of Units**	
Nursing monitoring	£113.00	14	PSSRU - Hospital-based nurses - Band 6 - cost per hour of patient contact = £113. Two contact hours per day for 7 days were assumed.[11]
Verapamil	£0.02	7	One verapamil 40-mg tablet per day at £1.95 for a pack size of 84[15]
Antihypertensive treatment	£0.04	7	One ramipril capsule 1.25 mg per day at £1.15 for 28 capsules[15]
**Adverse Event**	**Adverse Event Cost**		**Source/Assumptions**
Nephrotoxicity	£58.20		C Micallef and D Enoch, Cambridge University Hospitals NHS Foundation Trust, personal communication
**Components**	**Unit Cost**	**Number of units**	
NaCl infusion prophylaxis	£3.77	7	NaCl infusion 1L (1,000 mL), Sodium chloride 0.9% infusion 1 litre Macoflex bags[15],Co-trimoxazole SmPC (2020)[16]
Daily electrolytes	£3.71	7	DAPS08 – Phlebotomy[14]
Abdominal ultrasound	£52.13	2	RD40Z - direct access - Ultrasound Scan with duration of less than 20 minutes, without Contrast[14]

Daily urine collection	£1.10	7	DAPS04 - Clinical Biochemistry[14]
Dialysis in 1% of patients	£147.26	7	Hospital Haemodialysis or Filtration, with Access via Haemodialysis Catheter, 19 years and over.[14]
**Adverse Event**	**Adverse Event Costs**		**Source/Assumptions**
Tachycardia	£1,135.88		Resuscitation Council (UK) (2015)[20]
**Components**	**Unit Cost**	**Number of Units**	
Nurse monitoring	£113.00	14	PSSRU - Hospital-based nurses - Band 6 - cost per hour of patient contact = £113. Two contact hours per day for 7 days were assumed.[11]
ECG	£60.91	2	IMAG - RD51A - direct access - Simple echocardiogram, 19 years and over[14]

Adverse Event	Adverse Event Costs		Source/Assumptions
Hepatotoxicity	£73.23		Grade 3 adverse events: EASL (2019)[21]; C Micallef and D Enoch, Cambridge University Hospitals NHS Foundation Trust, personal communication
**Components**	**Unit Cost**	**Number of Units**	
Liver function test	£1.76	5	DAPS - DAPS09 - Other Pathology Services[14]
Alanine aminotransferase test	£1.10	5	DAPS - DAPS04 - Clinical Biochemistry[22]
Aspartate aminotransferase test			
Total bilirubin test			
Alkaline phosphatase test			
International normalised ratio test	£2.79	7	DAPS - DAPS05 – Haematology[22]
Corticosteroids	£2.02	7	Dose of 1-2 mg/kg/day.[21] Grade 3 adverse events. Methylprednisolone 1 gram vial g = £17.30.[15] Dose = 1.5 mg/kg/day. Average patient weight of 77.91 kg from NHS Health Survey for England.[23]

BNF = British National Formulary; EASL = European Association for the Study of the Liver; ECG = electrocardiogram; NaCl = sodium chloride; NHS = National Health Service; NICE = National Institute for Health and Care Excellence; PSSRU = Personal Social Services Research Unit.

REFERENCES

1. Zhao YJ, Khoo AL, Tan G, Teng M, Tee C, Tan BH, et al. Network meta-analysis and pharmacoeconomic evaluation of fluconazole, itraconazole, posaconazole, and voriconazole in invasive fungal infection prophylaxis. Antimicrob Agents Chemother. 2016 Jan;60(1):376-86.
2. Cornely OA, Maertens J, Winston DJ, Perfect J, Ullmann AJ, Walsh TJ, et al. Posaconazole vs. fluconazole or itraconazole prophylaxis in patients with neutropenia. N Engl J Med. 2007 Jan 25;356(4):348-59.
3. Hahn-Ast C, Glasmacher A, Mückter S, Schmitz A, Kraemer A, Marklein G, et al. Overall survival and fungal infection-related mortality in patients with invasive fungal infection and neutropenia after myelosuppressive chemotherapy in a tertiary care centre from 1995 to 2006. J Antimicrob Chemother. 2010 Apr;65(4):761-8.
4. Cordonnier C, Pautas C, Maury S, Vekhoff A, Farhat H, Suarez F, et al. Empirical versus preemptive antifungal therapy for high-risk, febrile, neutropenic patients: a randomized, controlled trial. Clin Infect Dis. 2009 Apr 15;48(8):1042-51.
5. Hebart H, Klingspor L, Klingebiel T, Loeffler J, Tollemar J, Ljungman P, et al. A prospective randomized controlled trial comparing PCR-based and empirical treatment with liposomal amphotericin B in patients after allo-SCT. Bone Marrow Transplant. 2009 Apr;43(7):553-61.
6. Pagano L, Caira M, Nosari A, Cattaneo C, Fanci R, Bonini A, et al. The use and efficacy of empirical versus pre-emptive therapy in the management of fungal infections: the HEMA e-Chart Project. Haematologica. 2011 Sep;96(9):1366-70.
7. Horn D, Goff D, Khandelwal N, Spalding J, Azie N, Shi F, et al. Hospital resource use of patients receiving isavuconazole vs voriconazole for invasive mold infections in the phase III SECURE trial. J Med Econ. 2016 Jul;19(7):728-34.
8. Ceesay MM, Sadique Z, Harris R, Ehrlich A, Adams EJ, Pagliuca A. Prospective evaluation of the cost of diagnosis and treatment of invasive fungal disease in a cohort of adult haematology patients in the UK. J Antimicrob Chemother. 2015 Apr;70(4):1175-81.
9. Bruynesteyn K, Gant V, McKenzie C, Pagliuca T, Poynton C, Kumar RN, et al. A cost-effectiveness analysis of caspofungin vs. liposomal amphotericin B for treatment of suspected fungal infections in the UK. Eur J Haematol. 2007 Jun;78(6):532-9.
10. NICE. Fungitell for antifungal treatment stratification. Medtech innovation briefing. National Institute for Health and Care Excellence; 31 August 2017. Available at: https://www.nice.org.uk/advice/mib118/resources/fungitell-for-antifungal-treatment-stratification-pdf-2285963281484485. Accessed 2 February 2021.
11. Curtis L, Burns A. Unit Costs of Health and Social Care 2019. Unit Costs of Health and Social Care. PSSRU. University of Kent; 2019. Report No.: 978-1-911353-10-2.
12. Talent.com. Medical delivery driver average salary in United Kingdom. 2021. Available at: https://uk.talent.com/salary?job=medical+delivery+driver. Accessed 4 February 2021.
13. NICE. SepsiTest assay for rapidly identifying bloodstream bacteria and fungi diagnostics guidance [DG20]. National Institute for Health and Care Excellence; 19 February 2020. Available at: https://www.nice.org.uk/guidance/dg20/chapter/4-Outcomes#diagnostic-accuracy. Accessed 2 February 2021.
14. NHS. National cost collection. National schedule of NHS costs, year 2018-19, NHS trust and NHS foundation trusts. National Health Service; 2020. Available at: https://www.england.nhs.uk/national-cost-collection/#ncc1819. Accessed 14 December 2020.
15. International Institute for Health Care and Excellence. British National Formulary. 2020. Available at: https://bnf.nice.org.uk/. Accessed 14 December 2020.
16. Co-trimoxazole SmPC. Co-trimoxazole summary of product characteristics. 2020. Available at: https://www.medicinescomplete.com/#/content/bnf/_962447623. Accessed 16 October 2020.
17. GOV.UK. VAT rates. 2020. Available at: https://www.gov.uk/vat-rates. Accessed 9 November 2020.

18. NHS. Mycology Reference Centre Manchester (MRCM). National Health Service. Manchester University. NHS Foundation Trust; 2020. Available at: http://mrcm.org.uk/index.php/test-catalogue/. Accessed 16 October 2020.
19. NICE. Hypertension in adults: diagnosis and management. NICE Guideline NG136. National Institute for Health and Care Excellence; 28 August 2019. Available at: https://www.nice.org.uk/guidance/NG13. Accessed 27 January 2021.
20. Resuscitation Council (UK). Guidelines 2015. Adult tachycardia (with pulse) algorithm. 2015. Available at: https://www.resus.org.uk/sites/default/files/2020-05/G2015_Adult_tachycardia.pdf. Accessed 27 January 2021.
21. EASL. European Association for the Study of the Liver (EASL) clinical practice guidelines: drug-induced liver injury. J Hepatol. 2019 Jun;70(6):1222-61.
22. NHS. Laboratory medicine. National Health Services. York Teaching Hospital. NHS Foundation Trust; Revised 4 February 2021. Available at: https://www.yorkhospitals.nhs.uk/our-services/a-z-of-services/laboratory-medicine1/. Accessed 2 March 2021.
23. NHS. Health Survey for England. 2016 trend tables. National Health Service; 2016. Available at: https://digital.nhs.uk/data-and-information/publications/statistical/health-survey-for-england/health-survey-for-england-2016. Accessed 27 October 2020.
